# Bladder and rectum dose estimations on digitized radiographs for vaginal brachytherapy after hysterectomy

**DOI:** 10.1120/jacmp.v15i6.5033

**Published:** 2014-11-08

**Authors:** Winson Zhang, Sudershan K. Bhatia, Wenqing Sun, Joseph M. Modrick, Yusung Kim

**Affiliations:** ^1^ Department of Biomedical Engineering University of Iowa Iowa City IA; ^2^ Department of Radiation Oncology University of Iowa Iowa City IA USA

**Keywords:** vaginal cylinder, high‐dose‐rate brachytherapy, dose‐distance modeling, endometrial cancer, brachytherapy

## Abstract

The purpose of this study was to evaluate the feasibility of assessing bladder and rectal point doses, using orthogonal radiographs without treatment planning, for vaginal cylinder applicator (VC), high‐dose‐rate (HDR) vaginal cuff brachytherapy (BT) after hysterectomy. Thirty‐three VC HDR BT treatment plans from 31 postoperative endometrial cancer patients were retrospectively analyzed. Single‐channel VC with four differing diameters — 2.0 cm, 2.3 cm, 2.6 cm, and 3.0 cm — were analyzed. Dose‐distance modeling was performed to estimate bladder and rectal point doses by measuring distances on each orthogonal radiograph without treatment planning. The estimated doses were then compared with doses calculated on treatment planning system (TPS). Their percent (%) dose differences were recorded. Analysis was performed for each VC size, ICRU bladder and rectal points, and the closest rectal point. The estimated doses obtained from dose‐distance modeling displayed on average less than 2.5% difference when compared with TPS doses at ICRU bladder and rectal points for each VC size. Dose percent differences between estimated values and TPS values were on average 1.9% and 2.5% for ICRU bladder and rectal point, respectively, regardless of VC sizes. Dose‐distance modeling for closest rectal point presented on average 5.4% dose difference when compared with TPS values of all VC sizes. It was feasible to estimate rectal and bladder point doses by measuring distances on orthogonal radiographs without treatment planning. Percent dose differences were 2.5% less for both ICRU bladder and rectal points, regardless of VC sizes. The use of closest rectal point is not recommended for estimating rectal dose.

PACS number: 87.53.‐j, 87.53.Jw, 87.55.‐x, 87.55.D‐, 87.55dk

## I. INTRODUCTION

Endometrial cancer is the most common gynecological malignancy.^(1)^ The American Cancer Society estimates that about 52,630 new cases of endometrial cancer will be diagnosed in 2014 and about 8,590 women will die of this malignancy in the United States.^(1)^ Radiation therapy after hysterectomy is the standard treatment for stage I–IV endometrial cancer. In particular, high‐dose‐rate (HDR) vaginal cuff brachytherapy (BT) without external beam radiation therapy (EBRT) has been widely used due to its efficacy, especially low local recurrence with minimal toxicity, low morbidity, and favorable quality of life after treatment.^2^, ^3^, ^4^, ^5^, ^6^, ^7^ The most common applicator used for HDR BT is a vaginal cylinder (VC) with a single, central channel.^(8)^ The standard treatment planning in VC HDR BT is based on two‐dimensional (2D) orthogonal radiographs. VC HDR BT treatment is typically planned for the first implant, and delivered after 2D imaging validations on treatment day. According to the American Brachytherapy Society (ABS) guidelines,^(9)^ customized treatment plans can be created for the first implant and the same plans are used for all following fractions, assuming the implant geometry remains the same for each implant. The VC HDR BT prescription dose is conventionally specified to 0 mm or 5 mm depth from the surface of the VC.^(9)^ The 2D‐based planning isodose lines are generated using two lateral reference lines at either 0 mm or 5 mm depth, and are not specific to the patient's anatomy but depend on the VC size. Dwell times for each VC size can, therefore, be generated by scaling them so as to account for activity differences. Precalculated dwell times for each VC size are described as an alternative to VC HDR BT treatment planning. However, ABS guideline recommends that radiographs or other measurements be taken to ensure that VC is in the same position in the vagina, relative to the organs at risk (OARs), such as the bladder and the rectum.^(9)^ For this reason, radiographs are not a prerequisite for dosimetric purposes, but for the validation of VC implants, per ABS guideline.^(9)^

In this study, we evaluate the feasibility of assessing bladder and rectal point doses using only orthogonal radiographs without treatment planning for hysterectomy patients receiving adjuvant VC HDR BT for endometrial cancer. The proposed technique is expected to simplify the planning process and reduce the treatment time needed for endometrial cancer treatment.

## II. MATERIALS AND METHODS

### A. Patients & vaginal cylinder (VC) HDR BT

**Figure 1:**
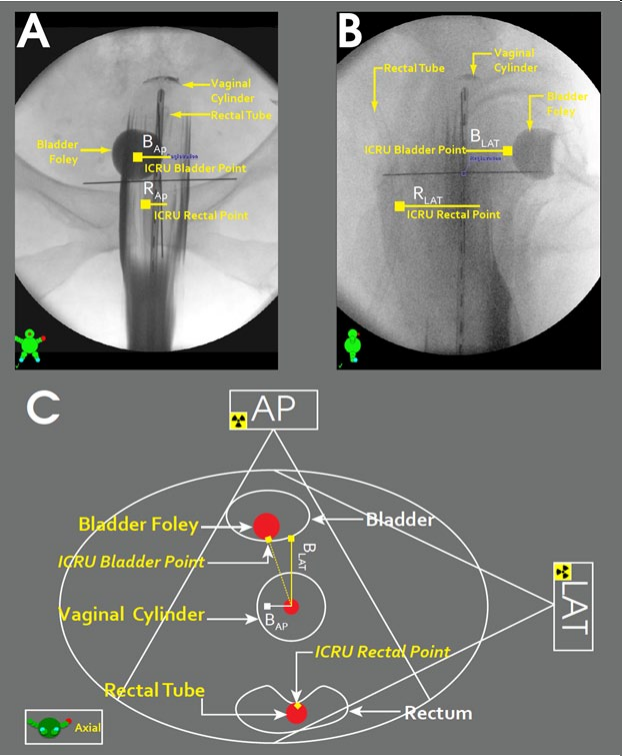
Anterior–posterior (AP) (a) and lateral (LAT) (b) view of an orthogonal radiograph set. Conceptualization of the corresponding axial view (c).

Thirty‐three VC‐HDR‐BT treatment plans from 31 biopsy‐proven, postoperative endometrial cancer patients were retrospectively studied after approval from our Institutional Review Board. Single‐channel VC (Varian Medical Solution, Inc., Palo Alto, CA) with differing diameters 2.0 cm (VC‐2.0cm), 2.3 cm (VC‐2.3cm), 2.6 cm (VC‐2.6cm), and 3.0 cm (VC‐3.0cm) were analyzed. The number of plans treated using each VC is listed in Table 1. Patients were treated with a fractionation scheme of 3 fractions of 6 Gy without EBRT with a prescription dose of 6 Gy delivered to a 5 mm depth from the VC surface at the vaginal apex. Orthogonal radiographs were obtained from a C‐arm fluoroscopy unit (ARCADIS Orbit, Siemens Medical Solutions, Inc., Erlangen, Germany) at the first implant, and were used as implant validation for the 2nd and 3rd fraction (Figs. 1(a) and (b)). A standard Foley catheter with a 7 cc of saline bulb and a rectal tube were inserted before placement of the VC applicator (Figs. 1(a) and (b)). The active dwell‐position length was 5 cm. Planning was performed using the BrachyVision version 8.9 (Varian Medical System, Inc.) treatment planning system (TPS) for optimization of treatment dose and calculation of ICRU (International Commission on Radiation Units and Measurements) bladder and rectal point doses.

**Table 1:** Mean dose %‐differences and other statistical analyses for ICRU bladder, rectal, and closest rectal points using dose‐distance modeling.

*VC‐Diameter*	*#. of Plans*	*Mean (SD) (%)*	*Min (%)*	*Max (%)*	$R^{2}$
*VC‐2.0cm*	8				
ICRU Bladder Point		2.5 (2.0)	0.7	5.7	0.99
ICRU Rectal Point		3.3 (2.0)	0.1	4.9	0.99
Close Rectal Point		4.4 (2.4)	0.1	5.4	0.93
*VC‐2.3cm*	7				
ICRU Bladder Point		1.1 (1.0)	0.1	3.1	0.99
ICRU Rectal Point		2.0 (1.0)	0.6	3.6	0.99
Close Rectal Point		9.3 (4.6)	2.9	14.0	0.80
*VC‐2.6cm*	10				
ICRU Bladder Point		2.1 (2.1)	0.3	7.2	0.98
ICRU Rectal Point		1.8 (1.0)	0.9	4.2	0.99
Close Rectal Point		2.1 (2.2)	0.5	7.3	0.97
*VC‐3.0cm*	8				
ICRU Bladder Point		1.7 (0.8)	0.7	2.8	1.0
ICRU Rectal Point		2.8 (2.1)	0.1	7.0	0.99
Close Rectal Point		5.9 (4.3)	1.2	12.1	0.96

SD = standard deviation; $R^{2}=\;\text{correlation coefficient}$.

### B. Reference points in dose‐distance modeling

Five and six points were clinically digitized on the rectal tube and bladder Foley balloon, respectively, to monitor their doses. For this study, three reference points (point doses) were analyzed with dose‐distance modeling, namely: the ICRU bladder point, ICRU rectal point, and closest rectal point. Conventional ICRU bladder and ICRU rectal points were defined on the radiograph (Figs. 1(a) and (b)) based upon the ICRU Report #38.^(10)^ The ICRU bladder point was defined as the posterior point of the Foley balloon as seen on the sagittal radiograph and the central point of the Foley balloon on the anterior–posterior (AP) radiograph. The ICRU rectal point was defined by drawing an anterior–posterior line from the lower end of the source to the rectal marker tube on the sagittal radiograph and was defined by drawing medial–lateral line from the lower end of the source to the rectal marker tube on the AP radiograph. The rectal wall or vaginal wall is not clearly visible on the radiographic image. The closest rectal point that is not shown on Fig. 1 was defined as the point on the rectal tube closest to the VC applicator. The radial distance is measured on orthogonal radiographs without treatment planning. To calculate the radial distance from the ICRU bladder, the ICRU rectal, or closest rectal points to VC on the orthogonal radiographs, the orthogonal distance in the AP and LAT images was measured and the radial distance determined from:

(1) $$d=\sqrt{d_{\text{AP}}{\;}^{2}+d_{\text{LAT}}{\;}^{2}}$$

An axial view of the radial distance is shown in Fig. 1(c).

### C. Dose‐distance modeling & dosimetric comparison

**Figure 2:**
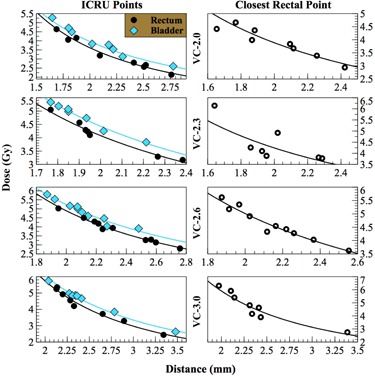
Dose‐distance modeling on four vaginal cylinder (VC) diameters.

Dose‐distance modeling is based on the inverse square law that states that the dose is inversely proportional to the square distance from the source. Once the distance from the reference point of interest (e.g., bladder point) to the applicator source is determined, dose‐distance modeling can be used to determine the dose absorbed at that particular point. We obtained the dose‐distance models by fitting the distances and the TPS doses to a power of −2 (inverse square law). This fitting was performed on all three reference points for all four predetermined VC sizes. A total of 12 models were generated, as shown in Fig. 2. To evaluate the feasibility of dose‐distance modeling, the estimated doses obtained from the dose‐distance modeling were compared with the TPS doses for all 12 models. Their dose percent differences (%‐difference) were recorded.

## III. RESULTS

The estimated doses (as seen in Fig. 2) obtained from the dose‐distance modeling displayed on average less than 2.5% difference when compared with TPS doses at ICRU bladder and rectal points for each VC size. Dose %‐differences between estimated values and TPS values were on average 1.9% and 2.5% for ICRU bladder and rectal point, respectively, regardless of VC sizes. The correlation coefficients (also seen in Table 1) between estimated doses and TPS doses were as high as 0.98, regardless of VC sizes. For ICRU bladder point, dose %‐difference was minimum (1.1%±1.0%) for VC‐2.3cm and maximum (2.5%±2.0%) for VC‐2.0cm. As for ICRU rectal point, dose %‐difference was minimum (1.8%±1.0%) for VC‐2.6cm and maximal (3.3%±2.0%) for VC‐2.0cm. The VC‐2.0 that had smallest number (i.e., eight) of cases for analysis has the greatest overall dose %‐differences: 2.5%±2.0% and 3.3%±2.0% for ICRU bladder and rectal point doses, respectively. Table 1 also demonstrates that the ICRU defined rectal point is more accurate and reliable in estimating the rectal dose than the closest rectal point. When using the closest rectal point, the average dose %‐difference increased from 2.5% to 5.4%. VC‐2.3cm presented a maximum dose %‐difference at 9.3%±4.6%.

## IV. DISCUSSION

The need for full treatment planning and routine dose calculation of OARs for use with single‐channel VC HDR BT without EBRT has been criticized for two main reasons: 1) the isodose lines of a single‐channel VC depends solely on the VC size; the isodose lines are not customized according to a patient's anatomy; and 2) the relatively low dose delivered to the OARs^(9)^ by single‐channel VC HDR BT when compared to EBRT and the overall low toxicity observed in single‐channel VC HDR BT.^(3)^ Evidence of favorable clinical outcomes persists, especially an improved survival rate and a quicker treatment recovery time when using single‐channel VC HDR BT.^2^, ^4^, ^5^, ^6^, ^7^ In particular, a simulation study by Barney et al.^(3)^ evaluating rectal and bladder toxicity using a single‐channel VC HDR BT reported that less than 5% of the patients (1 out of 24) developed a Grade 3 or higher toxicity level. In this study, we proposed using a dose‐distance modeling technique to estimate the OARs doses by measuring the distances between single‐channel VC and the ICRU rectum or the bladder point on orthogonal radiographs. The isodose lines for each single‐channel VC size can be generated by scaling its standard dwell times according to the activity level without having to complete the full treatment planning process. Since C‐arm radiographs are available in the majority of clinics, the rectum and bladder dose estimation technique using dose‐distance modeling could reduce time and resources spent on treatment planning. The proposed OARs dose estimation technique would be most beneficial to the large patient volume, a shortage of physicians, and in some cases both. The most obvious limitation to the widespread application of the proposed technique is that it is limited to single‐channel VC.

Conventionally, dose estimation techniques, such as nomograms,^(11)^ are used for quality assurance in BT treatment. A nomogram is used to estimate the number of seeds required for each implant or dose calculation in low‐dose‐rate prostate BT.^11^, ^12^ Kubo^(13)^ reported using simple mathematical formulas for checking single‐catheter BT treatment doses when prescribed at distances of 7.5 or 10 mm from the catheter center. Kubo found that the total treatment time was within ±2% for the 10 mm dose prescription.^(13)^ However, OARs dose estimation using radiographs for single‐channel VC HDR BT have rarely been reported. We found that the estimated ICRU rectal and bladder doses obtained through dose‐distance modeling matched the doses calculated using traditional treatment planning methods to within 2.5% difference. The American Association of Physicists in Medicine (AAPM) Task Group Report 56^(14)^ recommended that computer‐assisted dose calculations should have a numerical accuracy of at least ±2%. AAPM Task Group Report 40^(15)^ reported that an uncertainty of ±15% during the intracavitary delivery of BT is a realistic level of accuracy and precision in the delivery of prescribed dose. For the patients who do not present a high risk of OARs complications, the use of standard dwell times, instead of the full treatment planning process, can be used when rectum and bladder doses are estimated using dose‐distance model methods on orthogonal images. The clinical outcome difference analysis between full treatment planning and the use of dose‐distance modeling is outside the scope of this study. For single‐channel VC HDR BT, clinicians monitor rectal and bladder doses, but rarely change prescription dose because of the high doses occurring at the rectum or the bladder. This is due to the common perception that the clinical outcomes of single‐channel VC HDR BT will remain unaffected. By contrast multichannel VC^16^, ^17^ requires 3D imaging such as computed tomography (CT) and the use of volume optimization during treatment planning. The Capri applicator (Varian Medical Solution, Inc.),^16^, ^17^, ^18^ Novi Sad applicator (Nucletron, Inc., Veenendaal, The Netherlands),^(19)^ Vaginal CT‐MR Multi Channel applicator (Nucletron, Inc.),^17^, ^20^ and the CT/MR Miami MultiChannel Vaginal/Cervical applicator (Mick Radio‐Nuclear Instruments, Inc., New York, NY) are examples of commercially available multichannel VC applicators. Frontier studies used in‐house multichannel VC applicators.^21^, ^22^, ^23^ Due to an isotropic emission from a single Iridum‐192 source, further radiation dose sparing in OARs is often linked with a dose reduction to the vagina, consequently failing to deliver the prescribed dose. A multichannel cylinder enables clinicians to achieve sufficient dose reduction to OARs without compromising tumor coverage or to achieve the nonisotropic target coverage without overdosing the OARs.^22^, ^23^ For patients who are at increased risk of OAR toxicity, CT‐based multichannel VC HDR BT planning can offer more conformal radiation coverage for nonisotropic targets while sparing the rectum and the bladder under the recommended limits.^16^, ^17^, ^18^ A multichannel VC requires the use of 3D image, contouring of target and OARs, and a sophisticated treatment planning including each channel reconstruction and volumetric inverse planning.^(21)^ In addition, 3D image scans require that the multichannel VC HDR BT patient be transferred from the HDR treatment room to the CT or MR scanning room, unless the scanners are also present in the HDR treatment room. The complicated workflow and extended treatment planning procedures increases the overall multichannel VC HDR BT treatment time.^(21)^ For patients who are at an increased risk of toxicity due to higher a dose to OARs, we recognize that customized treatment planning technique using a CT‐based multichannel VC HDR BT technique would be preferable.

One of the limitations of this study is the small number of cases, especially for VC‐2.0cm size. The findings from this study should be confirmed by followup large‐scale studies.

We found that ICRU defined rectal point presented smaller percent differences than that of closest rectal point when comparing against rectal doses calculated using the TPS. This is partly because the ICRU reference point is relative in position only to the VC applicator, which is chosen to fit tightly within the vaginal canal so to eliminate air gaps,^(4)^ while the closest rectal point depends solely on the anatomical position of the rectum, and mostly varies between two patients even in VCs with a same diameter.

## V. CONCLUSIONS

Dose‐distance modeling is a feasible technique to assess ICRU bladder and rectal point doses on orthogonal radiographs without treatment planning. The estimated doses obtained from dose‐distance modeling display on average a difference of less than 2.5% when compared with treatment planning system doses at ICRU bladder and rectal points for each vaginal cylinder size. The use of closest rectal point is not recommended for estimating rectal dose.
